# Proteasomes in Patient Rectal Cancer and Different Intestine Locations: Where Does Proteasome Pool Change?

**DOI:** 10.3390/cancers13051108

**Published:** 2021-03-05

**Authors:** Pavel A. Erokhov, Alexey M. Kulikov, Yaroslava D. Karpova, Grigory V. Rodoman, Ilia R. Sumedi, Artem L. Goncharov, Dmitry V. Razbirin, Vera S. Gorelova, Natalia P. Sharova, Tatiana M. Astakhova

**Affiliations:** 1 Koltzov Institute of Developmental Biology of Russian Academy of Sciences, 26 Vavilov Street, 119334 Moscow, Russia; paer@freemail.ru (P.A.E.); a.m.kulikov@idbras.ru (A.M.K.); yasiiik@bk.ru (Y.D.K.); vsg57@bk.ru (V.S.G.); tastakhova@bk.ru (T.M.A.); 2 Pirogov Russian National Research Medical University of Ministry of Health of Russian Federation, 1 Ostrovityanov Street, 117997 Moscow, Russia; prof.rodoman@bk.ru (G.V.R.); soumedi@yandex.ru (I.R.S.); gonharov_artem@bk.ru (A.L.G.); d.razbirin@bk.ru (D.V.R.)

**Keywords:** patient rectal adenocarcinoma, rectal cancer surgery, proteasome activities, immune proteasomes, proteasome regulators, multiple proteasome forms, native electrophoresis

## Abstract

**Simple Summary:**

One of the most complicated problems during rectal cancer surgery is to determine what part of intestine to remove along with tumor. If the excised fragment will be too small, it could increase the risk of tumor recurrence. On the contrary, the removal of extra tissues could be completely unnecessary and complicate patient life a lot, especially when the region is close to sphincter. To determine the length of fragments to remove, it was necessary to reveal areas without changes in molecule functioning, specific for tumor. We investigated functioning the proteasomes, important cellular components, in patient rectal cancer and different intestine locations and did not reveal tumor-specific characteristics of proteasomes at sphincter side of intestine even at a distance of 2–4 cm from the tumor. This result supports the idea of preserving the sphincter under surgery. Besides, we discovered proteasome subtype that may be a promising target for anti-cancer therapy.

**Abstract:**

A special problem in the surgery of rectal cancer is connected with a need for appropriate removal of intestine parts, along with the tumor, including the fragment close to the sphincter. To determine the length of fragments to remove, it is necessary to reveal areas without changes in molecule functioning, specific for tumor. The purpose of the present study was to investigate functioning the proteasomes, the main actors in protein hydrolysis, in patient rectal adenocarcinoma and different intestine locations. Chymotrypsin-like and caspase-like activities, open to complex influence of different factors, were analyzed in 43–54 samples by Suc-LLVY-AMC- and Z-LLE-AMC-hydrolysis correspondingly. Both activities may be arranged by the decrease in the location row: cancer→adjacent tissue→proximal (8–20 cm from tumor) and distal (2 and 4 cm from tumor) sides. These activities did not differ noticeably in proximal and distal locations. Similar patterns were detected for the activities and expression of immune subunits LMP2 and LMP7 and expression of 19S and PA28αβ activators. The largest changes in tumor were related to proteasome subtype containing LMP2 and PA28αβ that was demonstrated by native electrophoresis. Thus, the results indicate a significance of subtype LMP2-PA28αβ for tumor and absence of changes in proteasome pool in distal fragments of 2–4 cm from tumor.

## 1. Introduction

The study of molecular mechanisms of malignant tumor development remains one of the acute problems thus far. A special place among oncological diseases is occupied by rectal cancer due to a need for appropriate surgical removal of intestine fragments from both sides of the tumor. The most problematic issue is determining the length of the fragment to remove at the distal part close to the sphincter. Solving this issue is tightly related to the detection of intestine areas where there are no changes in the molecule functioning that are intrinsic for tumor. In this regard, we paid attention to proteasomes, the main actors in protein hydrolysis, which largely determine the tumor fate [1,2,3,4]. Proteasomes control cellular proteome and regulate different processes by protein degradation and production of biologically active polypeptides and peptides.

The multiple functions of proteasomes are due to the multiplicity of their forms, which differ in the structure and specificity of the hydrolysis of protein substrates. According to the set of proteolytic subunits, proteasome pool in mammalian cells may be divided into constitutive (or housekeeping) proteasomes and subtypes of immune proteasomes [5]. The constitutive proteasomes contain proteolytic subunits β1, β2, and β5 in each of the two inner rings of their four-ring structure. These subunits exhibit caspase-like (CL), trypsin-like, and chymotrypsin-like (ChTL) activities, respectively. The immune proteasomes integrate immune proteolytic subunits – low molecular mass protein 2 (LMP2) (β1i), multicatalytic endopeptidase complex-like 1 (MECL1) (β2i), and LMP7 (β5i) instead of subunits β1, β2, and β5, respectively, in the process of new proteasome assembling. In the immune proteasomes, the conformation of substrate-binding pockets is modified so that the preferences for cleavage sites are changed and, therefore, the structure of produced peptides is changed too [6]. As a result, the immune proteasomes display high trypsin-like and ChTL activities and low CL activity and produce several times more epitopes for the major histocompatibility complex (MHC) class I molecules in comparison with constitutive ones. Hence, the immune proteasomes are important players in the immune response at several stages [7]. Interestingly, the immune subunits can be embedded in proteasomes either together or in various combinations with the constitutive subunits [8,9,10,11].

Besides, proteasome structures differ in activators (19S, PA28αβ/γ, PA200), which open the entry to proteasome proteolytic chamber for definite substrates [5]. As a rule, 19S activator recognizes ubiquitinated full-size protein substrates, while other activators help to utilize intermediate-size polypeptides without preliminary ubiquitination. The damaged/oxidized proteins can be processed by 20S core proteasome alone [12].

A few papers describe the expression of PSMB8 (LMP7 gene) [13], PA28γ activator [14], Rpt4 subunit of 19S activator [15] and possible clinical significance of these components of proteasome system in patient intestine cancers. In our previous work, we detected the enhanced ChTL activity as well as increased LMP7 and LMP2 content in rectal cancer and the decrease in these proteasome indicators after neoadjuvant chemoradiation therapy almost to their level in control tissue in majority of patients [16]. We proposed the possible use of these proteasome indicators to predict the effectiveness of neoadjuvant chemoradiation therapy in rectal cancer patients.

At the same time, a more detailed description of proteasome pool in rectal cancer and different intestine parts is necessary both for better understanding mechanisms of this disease development and for appropriate clinical outcomes. The main aim of the present study was to investigate proteasome functioning: activities of the total proteasome pool, expression of proteasome immune subunits and activators as well as activities of native proteasome subtypes in patient rectal cancer and different intestine fragments at the proximal and distal sides of the tumor.

## 2. Results and Discussion

### 2.1. Proteasome Activities in Rectal Cancer and Different Intestine Locations In Vitro

We investigated patient rectal adenocarcinoma, adjacent tissue, distal intestine fragments with the location of 2 cm and 4 cm from the tumor and proximal fragments with the location of 8 cm from the tumor (54 samples each), 15 cm (48 samples) and 20 cm (43 samples) from the tumor. The disease stages of patients (54% women and 46% men) were I, IIA–IIC and IIIA–IIIC (T_2–4b_N_0–2b_M_0_).

Proteasome ChTL and CL activities, open to the complex influence of different factors including the presence of one or another activator in the proteasome structure, combination of the proteasome proteolytic subunits, stress conditions, cellular status etc., are the best indicators of proteasome system state. Therefore, we focused our attention on the investigation of these proteasome activities. Besides, we studied the activities of LMP7 and LMP2 immune subunits which contributed to the total ChTL and CL activities correspondingly and reflected a functional state of the immune proteasomes. For detailed statistical analysis, we provide designation to proteasome activities in multiple intestine locations (Table 1).

Distribution normality of four proteasome activities in the tumor and six intestine locations in women and men with three disease stages was examined by Kolmogorov-Smirnov test, Lilliefors test and Shapiro–Wilk test (Tables S1–S6). Majority of distributions kept the symmetry indicating the stability on the evaluation by the variance analysis [17]. Overall, the data corresponded to multivariate normal distribution. The variance analysis is stable on assumption of dispersion equality violation [17]. In our investigation, absolute majority of distribution comparisons did not demonstrate significant violations of the equality (Tables S7–S9). Thus, the data obtained might be analyzed by standard methods of parametric statistics. Statistical indicators of the dependence of proteasome activities on the location in the intestine are shown in Table 2.

**Figure 1:**
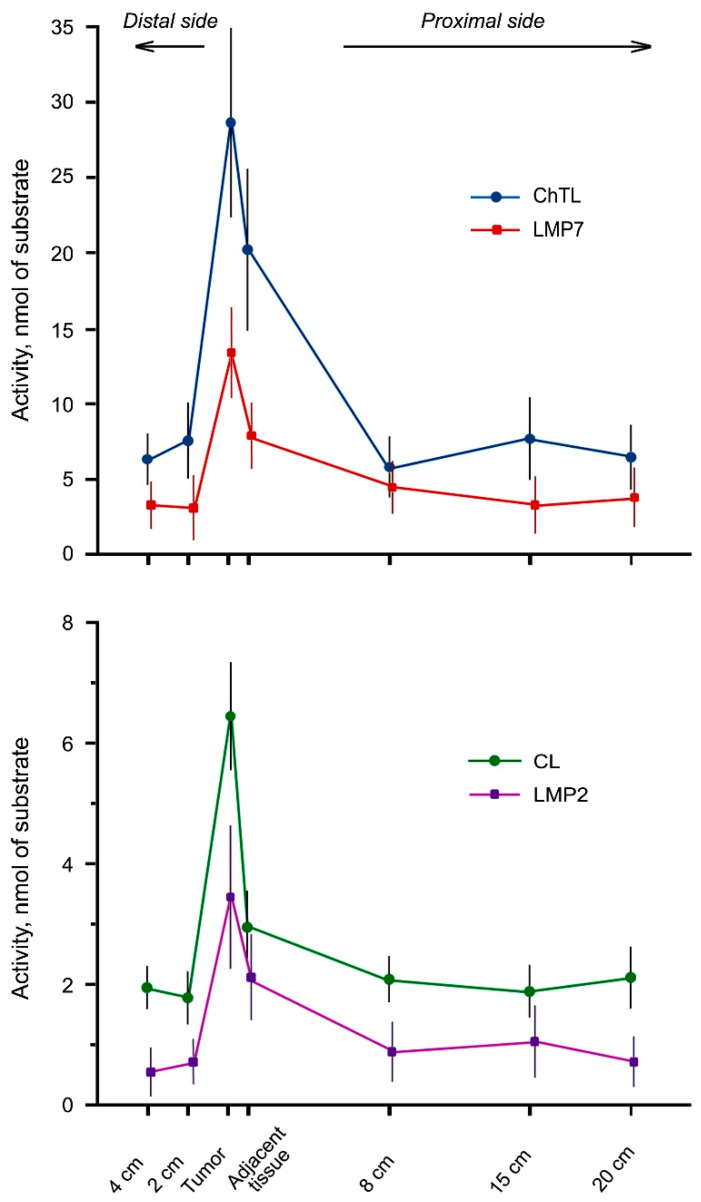
Proteasome ChTL, CL, LMP7 and LMP2 activities in rectal adenocarcinoma and different intestine parts of patients. The activities are expressed in nmol of substrate, hydrolyzed by 100 μL of clarified homogenate for 20 min. Standard deviation is shown as whiskers. The activities in the tumor and adjacent tissue differ reliably from the activities in other intestine locations at *p* < 0.001.

All studied proteasome activities were much higher in the tumor and adjacent tissue compared to other intestine locations (Figure 1). The activities may be arranged by the decrease in the location row: cancer→adjacent tissue→proximal and distal intestine sides. Interestingly, in the proximal and distal intestine parts the form of ChTL activity plot is similar to the form of LMP2 activity plot, while the form of CL activity plot is similar to the form of LMP7 activity plot (Figure 1). At first sight, these results are contradictory as LMP2 subunit, along with β1, displays CL activity, and LMP7 subunit, along with β5, displays ChTL activity [5]. This apparent contradiction may be explained if LMP2 and LMP7 subunits are mainly embedded in different proteasome structures, containing LMP2–β5 and LMP7–β1 combinations. At the same time, a lesser part of proteasomes may include LMP2–LMP7 combination.

Although each of the four proteasome activities did not differ markedly in the distal and proximal intestine parts (Figure 1), we used homogeneity tests for a finer analysis (Table 3 and Table 4). By Fisher LSD and Bonferroni tests, all studied activities in the tumor and adjacent tissue occupied independent clusters. For ChTL, LMP7 and LMP2 activities, Bonferroni test combined the remaining values in the common cluster, while LSD test divided them into two overlapped clusters. At the same time, both tests divided CL activity in the distal and proximal intestine parts into two/three overlapped clusters (Table 3). In general, these tests showed that despite belonging to overlapping clusters, the activities in separate distal parts of the intestine were lower than the activities in the majority of proximal parts and therefore they differed most from the values in the tumor.

For correlation analysis, we took into account significance level *p* ≤ 0.01. The most significant correlation was related to ChTL activity (Table 5), which was the highest among other activities (Figure 1). Correlated ChTL activity belonged to topologically close areas including tumor, adjacent tissue and distal fragments of 2 and 4 cm from the tumor as well as to proximal fragments of 15 and 20 cm among themselves. It is interesting that ChTL activity in the most distant proximal fragments (15 and 20 cm) correlated with ChTL activity in the distal parts close to the tumor (2 and 4 cm).

All these results indicate rather different cellular composition of distal and proximal intestine parts than the presence of pathological phenomena. For example, some intestine fragments may contain cells of the immune system. Thus, the intestine distal area even in 2–4 cm from the tumor is likely to be normal by proteasome activities. This fact along with other data to be collected may be important for the intestine surgery.

Variance factorial analysis was carried out for the whole multitude of dependent factors (proteasome activities and location) taking into account a possible influence of grouping factors (gender and disease stage). Overall, factors of gender and disease stage did not influence proteasome activities (Table S10).

Thus, different statistical tests showed that significant changes in all studied proteasome activities concerned rectal cancer and adjacent tissue.

### 2.2. Expression of Proteasome Pool Components and MHC Class I Molecules in Rectal Cancer and Different Intestine Locations

**Figure 2:**
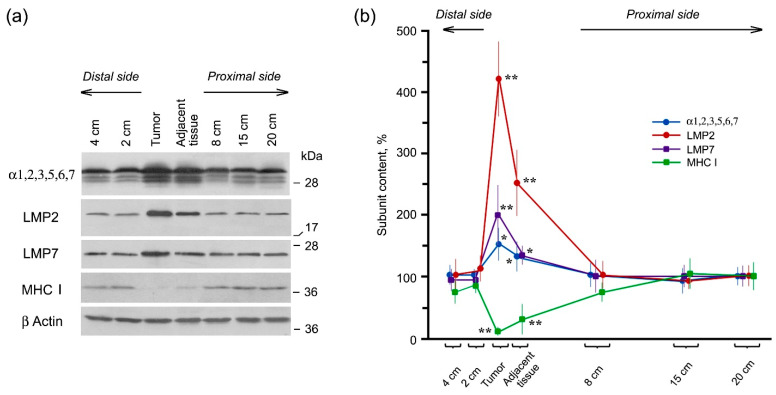
Content of proteasome subunits and MHC class I molecules in rectal adenocarcinoma and different intestine parts of patients. (**a**) Western blots of polypeptides in cleared homogenates with the use of the corresponding antibodies. Molecular mass of standard protein markers is shown. (**b**) The relative polypeptide content normalized to β actin level in percentage from the content in the most distant tissue sample (20 cm from the tumor). Standard deviation is shown as whiskers. The content of all polypeptides in the tumor and adjacent tissue differs reliably from that in other intestine locations at *p* < 0.05 (*) and *p* < 0.001 (**).

To explore whether a dramatic increase in the proteasome activities in the tumor is influenced by altered proteolytic subunit composition and/or specific activator presence, we studied the content of proteasome subunits and activators on the protein level by Western blotting. The specificity of the antibodies used is shown in full-size images of gels reflected on X-ray films (Figures S1 and S2). The expression of immune LMP2 and LMP7 subunits as well as α1,2,3,5,6,7 subunits (marker of the total proteasome pool) was higher in the tumor and adjacent tissue in comparison with distal and proximal intestine parts similar to the proteasome activities (Figure 2).

Note, LMP2 expression increased 4-fold while LMP7 expression increased only 2-fold in the tumor in comparison with control. Most likely, this difference results from the embedding of a larger amount of LMP2 subunit in newly formed proteasome structures without LMP7 subunit. High levels of the immune proteasomes are essential for the formation of foreign antigenic epitopes including cancer epitopes and subsequent development of the immune response. However, rectal cancer cells expressing the high levels of the immune proteasomes avoid the immune surveillance. It is possible that cancer cells lack other components for the immune reaction development. We checked the availability of MHC class I molecules in the tumor. Complex of MHC class I molecules and antigen epitope serves as a marker on the cell surface for recognition by T-killers [7]. We revealed that the content of MHC class I molecules was drastically reduced in rectal cancer and adjacent tissue compared to other studied intestine locations (Figure 2). We could strongly suggest that despite the increased level of the immune proteasomes, rectal cancer cells avoid the immune surveillance due to the low content of MHC class I molecules. It is obvious that cancer cells have adapted immune proteasomes to their own needs.

**Figure 3:**
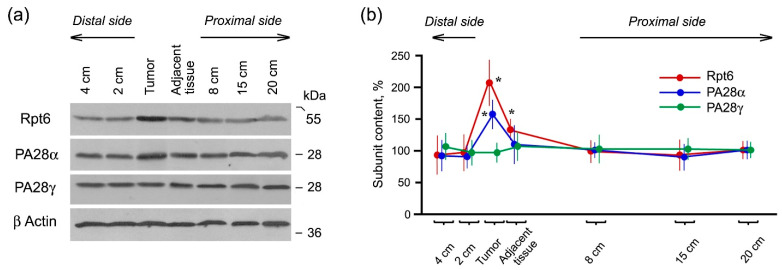
Content of proteasome activators’ subunits in rectal adenocarcinoma and different intestine parts of patients. (**a**) Western blots of subunits in cleared homogenates with the use of the corresponding antibodies. Molecular mass of standard protein markers is shown. (**b**) The relative subunit content normalized to β actin level in percentage from the content in the most distant tissue sample (20 cm from the tumor). Standard deviation is shown as whiskers. * The reliable difference from distal and proximal intestine locations at *p* < 0.01.

The expression of proteasome activators 19S, PA28αβ and PA28γ was studied with the use of the antibodies to Rpt6, PA28α and PA28γ subunits included in the composition of these activators, respectively. The expression of Rpt6 and PA28α subunits increased in the tumor (Figure 3).

The reason for the increased expression of 19S activator is clear, since it is involved in the maintenance of the enhanced protein metabolism in actively dividing malignant cells. In particular, enhanced ubiquitination and degradation of p53 were found in colorectal cancer cells [18]. However, what can be the role of PA28αβ activator in tumor cells? Previously, we detected that the high levels of PA28αβ activator and LMP2 (but not LMP7) subunit in allograft cells were associated with the allograft engraftment regardless of donor-recipient differences [19]. We assume that LMP2 subunit and PA28αβ activator embedded in the same proteasome structure, are involved in ensuring the survival of rectal cancer cells, foreign for an organism.

On the contrary, the expression of PA28γ activator did not reliably increase in the studied tumor samples. This result differs from the data published by D. Chen et al. on enhanced PA28γ expression in most of samples of colorectal cancer [14]. We think that such discrepancy is based on the difference of studied TNM stages. There were no tumors with initial T1 size in our investigation, while D. Chen and co-authors underlined the role of PA28γ in the early events of colorectal cancer. PA28γ activator is known to regulate nuclear proteolysis and cell proliferation [5]. Obviously, its role in starting steps of malignant cell division is more important.

Thus, we discovered that the increase in proteasome activities in rectal cancer was connected with the enhanced expression of the proteolytic LMP2 and LMP7 immune subunits as well as 19S and PA28αβ activators.

### 2.3. Proteasome Activities in Native Gel

**Figure 4:**
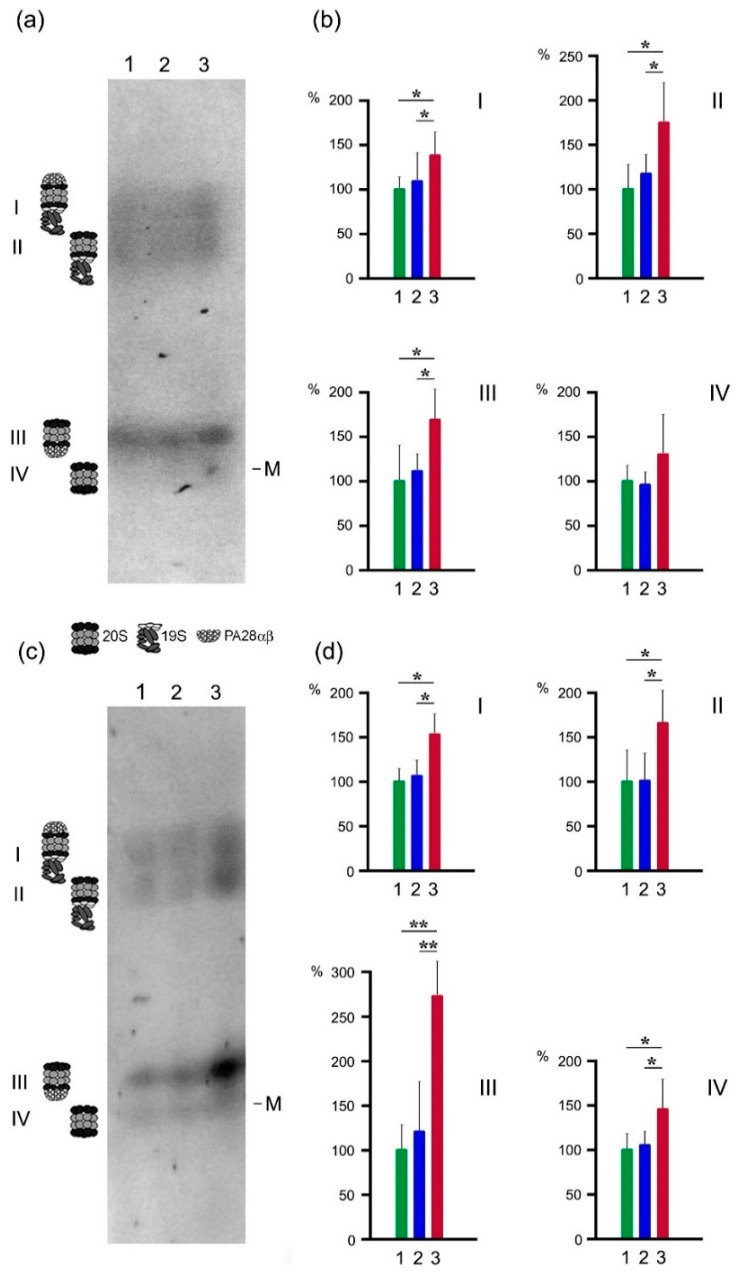
CL and LMP2 activities of proteasome subtypes in rectal adenocarcinoma and different intestine parts of patients. (**a**) CL activity in native PAG. (**b**) Relative CL activity for every gel zone. (**c**) LMP2 activity in native PAG. (**d**) Relative LMP2 activity for every gel zone. Thyroglobulin (670 kDa), labeled by dye Cy-3.5, was used as a marker (M) of molecular mass. Proteasome activity in intestine distal side in 2 cm from the tumor (taken as 100%) (1) and proximal side in 15 cm from the tumor (2) and tumor (3). Standard deviation is shown, *p* < 0.05 (*), *p* < 0.005 (**).

**Figure 5:**
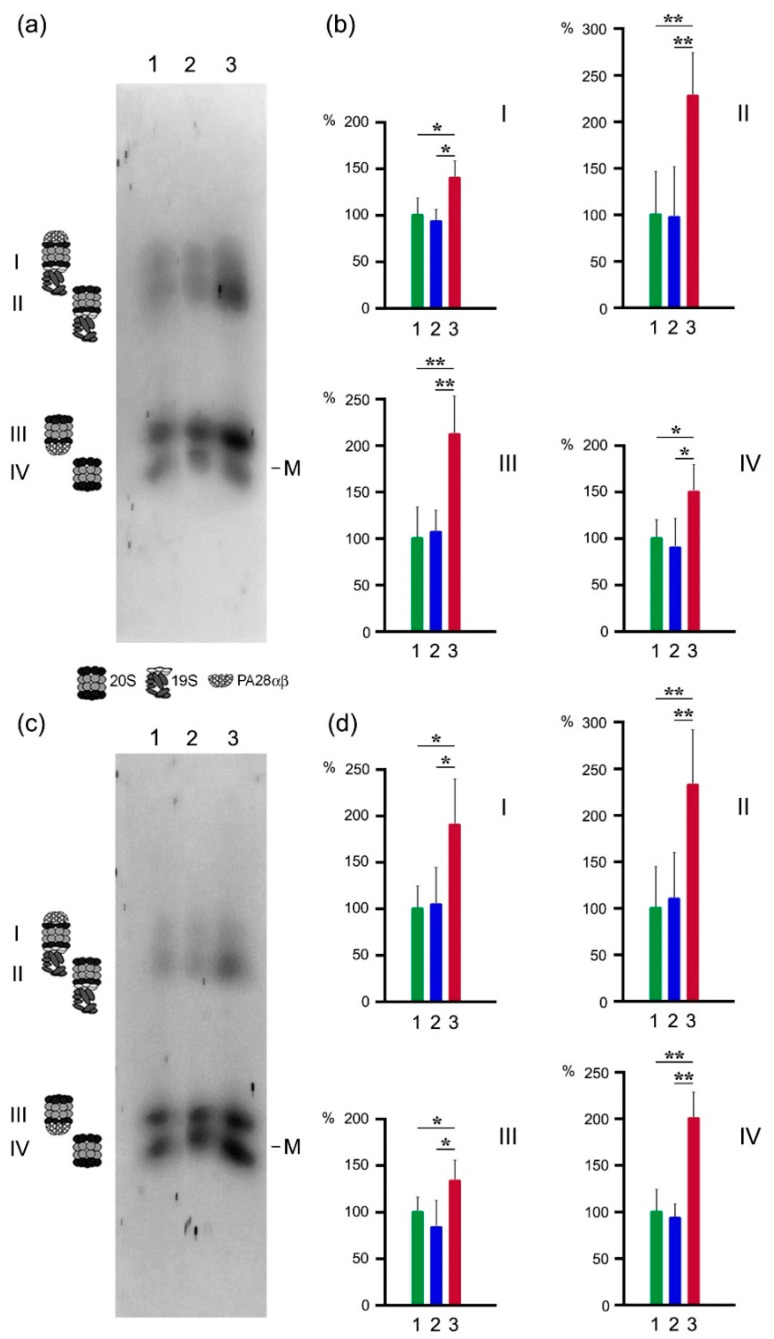
ChTL and LMP7 activities of proteasome subtypes in rectal adenocarcinoma and different intestine parts of patients. (**a**) ChTL activity in native PAG. (**b**) Relative ChTL activity for every gel zone. (**c**) LMP7 activity in native PAG. (**d**) Relative LMP7 activity for every gel zone. Thyroglobulin (670 kDa), labeled by dye Cy-3.5, was used as a marker (M) of molecular mass. Proteasome activity in intestine distal side in 2 cm from the tumor (taken as 100%) (1) and proximal side in 15 cm from the tumor (2) and tumor (3). Standard deviation is shown, *p* < 0.05 (*), *p* < 0.005 (**).

To check whether LMP2 subunit and PA28αβ activator belong to the same proteasome structure, we used the original native electrophoresis method, adapted for crude proteasome fractions [20] (Figure 4). This method is useful for mapping the activities of various proteasome forms. CL activity, related to β1 and LMP2 subunits, and LMP2 activity were detected in four gel zones, I–IV (Figure 4a,c). Zones I and II contain proteasomes with 19S activator, zone III contains proteasomes with PA28αβ activator, 20S core proteasome structures without any activator are located in zone IV [20]. ChTL activity, related to β5 and LMP7 subunits, and LMP7 activity were also found in gel zones I–IV (Figure 5a,c). Active proteasome zones in native gel look as wide strains, not as narrow stripes, since they cover diversity of the proteasome subtypes that distinguish at least in proteolytic subunits’ combination.

All studied activities were higher in the tumor compared to the intestine parts in all revealed proteasome structures. Note, the most essential enhance is related to LMP2 activity (by 175%) of the proteasome structures containing PA28αβ activator (gel zone III) (Figure 4c,d). In this gel zone, ChTL activity also increased significantly (by 115%) (Figure 5a,b), whereas CL and LMP7 activities increased only by 65% and 35% (Figure 4a,b and Figure 5c,d). These facts along with the results described above show that among the proteasomes newly formed in the tumor, the proteasome subtype containing proteolytic immune subunit LMP2, proteolytic constitutive subunit β5 (displaying ChTL activity) and activator PA28αβ, is dominant. In addition, the minor part of PA28αβ-containing proteasomes includes LMP7–LMP2 and/or LMP7–β1 combinations.

Besides, in gel zone II, ChTL and LMP7 activities were also noticeably increased (by 130%) in the tumor sample (Figure 5). At the same time, CL and LMP2 activities increased by 75% and 65%, respectively (Figure 4). Obviously, this gel zone comprises two major newly formed proteasome subtypes with 19S activator in approximately the same amounts. One subtype contains subunits LMP7 and β1 (displaying CL activity), the second subtype contains subunits LMP7 and LMP2. Embedding the immune subunits enhances proteasome ChTL, but not CL activity [5], and our results are consistent with these data. We have to emphasize that all revealed proteasome forms in gel zones I–IV display the activities of LMP2 and LMP7 subunits to a greater or lesser extent. Such multiplicity of the proteasome forms in rectal cancer may be required for the utilization of the diverse protein substrates.

It is intriguing that in other tumor types (patient thyroid papillary carcinoma, mouse hepatocellular carcinoma and mammary gland cancer) as well as in patient rectal cancer the increase in LMP2 subunit expression is much more significant in comparison with the expression of LMP7 subunit [2,21,22]. It indicates the universal role of the proteasomes with LMP2 subunit in the tumor development. By analogy with the role of LMP2 subunit and PA28αβ activator in the survival of allotransplant [19], we believe that these proteasome components are important for the survival of tumor cells. In the present study, we discovered that the great increase in LMP2 activity is related to proteasome subtype containing PA28αβ activator. On the basis of the data obtained we hypothesize that proteasome subtype LMP2–β5–PA28αβ produces small peptides which are released into the intercellular space to suppress the activity of T-killers. This process is performed without MHC class I molecules. Intermediate-size polypeptide substrates for LMP2–β5–PA28αβ proteasome are likely to be produced by proteasome subtypes with 19S activator from full-size proteins. Our hypothesis is supported by the results obtained by M. Raule et al. [23]. The authors discovered that 20S–PA28αβ proteasomes generated a smaller fraction of peptides with a length of 8–10 amino acid residues compared to 20S and 20S–19S proteasome forms. Taking into account that peptides of this particular length can serve as epitopes for MHC class I molecules, one can conclude that the major function of PA28αβ is not related to the formation of epitopes. Moreover, binding of PA28αβ to 20S particle dramatically reduced the overall efficiency of generation of peptides longer than 10 amino acids which might serve in MHC class I antigen presentation after appropriate trimming by aminopeptidases in the cytosol or endoplasmic reticulum [23]. In tumor cells, proteasomes containing PA28αβ activator and LMP2 proteolytic subunit seem to provide peptides of a special structure to maintain tumor survival and growth.

We conclude that LMP2 subunit alone or together with PA28αβ activator may be a promising target for anticancer therapy. However, we should remember about the necessity of address delivery of anti-LMP2 drug to tumor cells, since LMP2 subunit participates in many important processes including the immune response and adaptive reactions of the brain that must not be impacted [7,20]. This may indicate possible future strategies of antitumor therapeutics development that should be based upon principles of evident fundamental research and not merely on extensive multiplying proteasome inhibitors, a way pitifully taken as a basic principle by a lot of pharmaceutical manufacturers nowadays [24,25,26,27,28,29,30,31]. Based on the fundamental research, a number of investigators offer using subunits of proteasome 19S activator as targets for antitumor therapy [15,32,33,34]. Moreover, we have developed antitumor compositions inhibiting the functioning of 19S activator and proved their effectiveness and low toxicity in preclinical trials [34]. In the present study, we pay attention to LMP2-PA28αβ subtype as the other promising anticancer target in proteasome pool.

Another important finding of this work is the absence of tumor-like changes in the proteasome pool in the distal intestine fragments even at a distance of 2 and 4 cm from the tumor. This result supports the idea of preserving the sphincter under surgery.

## 3. Materials and Methods

### 3.1. Patient Tissue Samples

For the study, sampling was randomized among untreated Moscow patients with verified rectal adenocarcinoma surgically removed in Pirogov Russian National Research Medical University of Ministry of Health of Russian Federation since February 2015 to October 2019. The median age of the patients (54% women and 46% men) was 68 years old, ranging from 51 to 82 years. The disease stages were I, IIA–IIC and IIIA–IIIC (T_2–4b_N_0–2b_M_0_).

Anonymity was achieved by encrypting patient names with letter characters. Blinding was achieved by numeric designation of tumor and intestine tissue samples. Decoding was carried out after the experimental procedures.

As a result, we investigated 54 samples of the tumor, as well as 54 samples of the adjacent tissue (up to 0.7 cm from the tumor). Besides, we investigated 54 samples of the intestine distal fragment with the location of 2 cm from the tumor, 54 samples of the distal fragment with the location of 4 cm from the tumor, 54 samples of the proximal fragment with the location of 8 cm from the tumor, 48 samples of the proximal fragment with the location of 15 cm from the tumor and 43 samples of the proximal fragment with the location of 20 cm from the tumor. The tissue samples were stored at −80 °C during a month. For native electrophoresis, unfrozen tissues were used.

### 3.2. Antibodies and Main Reagents

Combined mouse monoclonal antibodies (mAb) to proteasome 20S α1,2,3,5,6&7 subunits (BML-PW8195), mouse mAb to proteasome 19S ATPase subunit Rpt6 (BML-PW9265), to proteasome 20S β1i (LMP2) subunit (BML-PW8840), to proteasome 20S β5i (LMP7) subunit (BML-PW8845), rabbit polyclonal antibodies to proteasome activator 11S α (PA28α) subunit (BML-PW8185), to proteasome activator 11S γ (PA28γ) subunit (BML-PW8190) were purchased from Enzo Life Sciences, New York, NY, USA; mouse mAb to β actin (ab8226), rabbit mAb to MHC class I + HLA B (ab110645) were purchased from Abcam, Cambridge, UK.

Proteasome ChTL activity substrate N-succinyl-leu-leu-val-tyr-7-amido-4-methyl coumarin (Suc-LLVY-AMC) (S6510), proteasome CL activity substrate Z-Leu-Leu-Glu-7-amido-4-methyl coumarin (Z-LLE-AMC) (C0483), proteasome inhibitor Z-leucyl-leucyl-leucinal (MG132) (C2211) were purchased from Sigma-Aldrich, St. Louis, MO, USA; proteasome β1i (LMP2) activity substrate Ac-Pro-Ala-Leu-AMC (Ac-PAL-AMC) (S-310), proteasome β5i (LMP7) activity substrate Ac-Ala-Asn-Trp-AMC (Ac-ANW-AMC) (S-320) were purchased from Boston Biochem, Cambridge, MA, USA.

### 3.3. Preparation of Clarified Tissue Homogenates

For the investigation of proteasome activities in vitro and Western blotting, clarified homogenates of the tumor and intestine fragments (the tissue separated from muscle layer) were prepared in six volumes (*w*/*v*) of buffer containing 50 mM Tris-HCl (pH 7.5), 200 mM NaCl, 1 mM EDTA, 1 mM dithiothreitol, 10% glycerin, 5 mM MgCl_2_, 1 mM ATP, 10 mM Na_2_S_2_O_5_, leupeptin (0.5 μg/mL), pepstatin (1 μg/mL) and aprotinin (1 μg/mL) as described previously [21].

For native electrophoresis, clarified tissue homogenates were prepared in three volumes (*w*/*v*) of buffer containing 50 mM Na-HEPES, pH 7.5, 200 mM NaCl, and 10 mM EDTA as earlier described [20].

### 3.4. Detection of Proteasome Activities In Vitro

Proteasome ChTL and CL activities were determined by the hydrolysis of fluorogenic substrates Suc-LLVY-AMC and Z-LLE-AMC correspondingly. Proteasome LMP7 and LMP2 activities were determined by the hydrolysis of fluorogenic substrates Ac-ANW-AMC and Ac-PAL-AMC correspondingly.

The reaction mixture contained 20 mM Tris-HCl (pH 7.5), 30 μM substrate, 1 mM dithiothreitol, 5 mM MgCl_2_, and 1 mM ATP. To calculate the contribution of non-proteasome proteolytic activities, 7 μM MG132 (proteasome inhibitor) was used. The reaction was carried out at 37 °C for 20 min after adding 0.5, 1.0, 1.5 and 2.0 μL of clarified homogenate (to a total volume of 100 μL) and terminated by 1% SDS. The product was detected by using a fluorimeter with the excitation wavelength 380 nm and the emission wavelength 440 nm. The difference between the total activity and residual activity in the presence of MG132 was determined as proteasome activity. The residual activity in the presence of MG132 did not exceed 10%. Under the indicated conditions, the quantity of the reaction product is proportional to reaction duration [20].

### 3.5. Western Blotting

After SDS electrophoresis in 13% polyacrylamide gel (10 μL of clarified homogenate or 90–105 μg of protein per lane), polypeptides were transferred from the gel onto nitrocellulose membrane by the standard procedure. Immunodetection was carried out with the use of primary antibodies to α1,2,3,5,6,7, LMP7, LMP2, MHC class I, β actin (1:1000), Rpt6, PA28α, PA28γ (1:1500), and corresponding secondary antibodies peroxidase conjugated (1:2000). The image analysis was performed using standard ImageJ software (an open-source software developed by contributors worldwide which is available at any time in the public domain distributed under BSD-2 licence https://imagej.net/Welcome). 

### 3.6. Detection of Proteasome Activities in Native Gel

Proteasome activities in native gradient 4–10% polyacrylamide gel (5 μL of clarified homogenate or 75–85 μg of protein per lane) was detected with the use of fluorogenic 300 μM substrates in 200 mM Na-HEPES buffer, pH 7.5 (1/20 of gel volume) after electrophoresis at 60 V for 14 h, 140 V for 10 h and 260 V for 20 h, as described previously [20]. Fluorescence bands in the gel were photographed under 365 nm UV light. The image analysis of proteasome activities in the gels was performed using the standard ImageJ software.

### 3.7. Technical Remarks

(1) We investigated the expression of LMP2 and LMP7 immune subunits, but not MECL1 subunit, since generally MECL1 and LMP2 subunits are mutually required for incorporation into 20S structure, with incorporation of MECL1 depends on LMP2 and incorporation of LMP2 is facilitated by MECL1 [8,9]. Therefore, the change of LMP2 content reflects the change of MECL1 content.
(2) For our investigation, the methods requiring proteasome purification, such as quantitative proteomics and similar techniques, were irrelevant, as different steps of proteasome purification lead to partial or total loss of one or another proteasome form and distortion of in-cell state [35,36]. We used the original method of electrophoresis in native gel, specially developed for crude proteasome fractions, to reflect in-cells state of “alive” proteasomes in tissues most correctly.

### 3.8. Statistics

Statistical treatment with the use of factorial ANOVA, post hoc tests (Fisher, Bonferroni, Newman-Keuls tests), correlation analysis was performed in Statistica 10. (StatSoft Inc., 2010, Tulsa, OK, USA; https://www.tibco.com, accessed on 15 December 2020). The observed power of ANOVA in all cases was 1.

## 4. Conclusions

**Figure 6:**
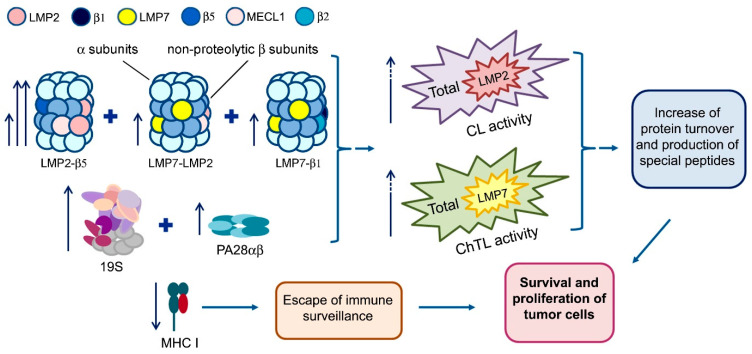
Scheme of changes in proteasome pool and MHC class I molecules’ content in patient rectal adenocarcinoma in comparison with conditionally normal intestine tissue.

In the present paper, we described the changes of proteasome pool in patient rectal adenocarcinoma in comparison with conditionally normal intestine tissue. The results obtained are briefly summarized in Figure 6.

The main changes in the tumor are following: (1) the formation of new proteasome structures with LMP2 subunit in quantity exceeding three times the initial level; (2) the formation of new proteasome structures with LMP7 subunit in approximately equal numbers compared to the initial level; (3) the expression of the additional quantities of 19S and PA28αβ activators; (4) the increase in the total proteasome pool; (5) the enhance of CL, ChTL, LMP2 and LMP7 activities, especially LMP2 activity in the structure, containing PA28αβ activator; (6) the decrease in the content of MHC class I molecules. These events lead to the intensive protein turnover, production of special peptides and survival and proliferation of tumor cells. The obtained results may be useful for a better understanding of proteasome mechanisms of the rectal cancer development. At the same time, the further study in this area is required. What peptides are produced by proteasome LMP2–β5–PA28αβ subtype and how can they participate in the suppression of the immune response? The aim of our subsequent investigation will be directed to solving these issues.

Besides, the present study has certain medical outcomes. First, the results indicate the LMP2-PA28αβ subtype as a promising target for anticancer therapy on condition that address delivery of anti-LMP2-PA28αβ drugs to tumor cells would be developed. Second, the results show that the distal intestine part close to the sphincter, even in 2–4 cm from the tumor, has no peculiarities of the proteasome pool specific for the tumor. These results, along with other facts to be collected, may be very important for preserving the sphincter under surgery intervention.

## Figures and Tables

**Table 1:** Conditional designation of factors “Proteasome activities in the intestine locations”.

Factors	Designation
Activities in tumor	(1)
Activities in adjacent tissue	(2)
Activities in intestine distal side, 4 cm from tumor	(3)
Activities in intestine distal side, 2 cm from tumor	(4)
Activities in intestine proximal side, 8 cm from tumor	(5)
Activities in intestine proximal side, 15 cm from tumor	(6)
Activities in intestine proximal side, 20 cm from tumor	(7)

**Table 2:** Dependence of proteasome activities on the location in the intestine.

Activity	Statistical Indicators		
	df	Fst	*p*
ChTL	6	276.9	<0.0001
CL	6	414.2	<0.0001
LMP7	6	77.1	<0.0001
LMP2	6	58.9	<0.0001

**Table 3:** Homogeneity of proteasome ChTL and CL activities in different intestine parts.

PL	Homogenous Groups of ChTL Activity							PL	Homogenous Groups of CL Activity								
	Fisher LSD Test				Bonferroni Test				Fisher LSD Test					Bonferroni Test			
	1	2	3	4	1	2	3		1	2	3	4	5	1	2	3	4
(5)		****			****			(4)	****					****			
(3)	****	****			****			(3)	****	****				****	****		
(7)	****	****			****			(6)	****	****	****			****	****		
(4)	****				****			(5)		****	****			****	****		
(6)	****				****			(7)			****				****		
(2)			****			****		(2)				****				****	
(1)				****			****	(1)					****				****

Here and in Table 4: ****, homogenous group; PL, proteasome location.

**Table 4:** Homogeneity of proteasome LMP7 and LMP2 activities in different intestine parts.

PL	Homogenous Groups of LMP7 Activity							PL	Homogenous Groups of LMP2 Activity						
	Fisher LSD Test				Bonferroni Test				Fisher LSD Test				Bonferroni Test		
	1	2	3	4	1	2	3		1	2	3	4	1	2	3
(4)	****				****			(3)	****				****		
(6)	****				****			(7)	****	****			****		
(3)	****	****			****			(4)	****	****			****		
(7)	****	****			****			(5)	****	****			****		
(5)		****			****			(6)		****			****		
(2)			****			****		(2)			****			****	
(1)				****			****	(1)				****			****

**Table 5:** Correlation of proteasome ChTL activity in tumor and different intestine parts.

PL	C and *p* Indicators for Different Proteasome Locations						
	(1)	(2)	(3)	(4)	(5)	(6)	(7)
(1)	1.0000	**0.6611**	**0.5466**	**0.6188**	0.3226	0.5058	0.4109
(2)	***p* = 0.001**	1.0000	**0.6368**	**0.5780**	0.4690	0.4859	0.4611
(3)	***p* = 0.010**	***p* = 0.002**	1.0000	**0.8615**	0.5296	**0.7880**	**0.6174**
(4)	***p* = 0.003**	***p* = 0.006**	***p* < 0.001**	1.0000	0.4418	**0.7709**	**0.6595**
(5)	*p* = 0.154	*p* = 0.032	*p* = 0.014	*p* = 0.045	1.0000	0.4611	0.4058
(6)	*p* = 0.019	*p* = 0.026	***p* < 0.001**	***p* < 0.001**	*p* = 0.035	1.0000	**0.8048**
(7)	*p* = 0.064	*p* = 0.035	***p* = 0.003**	***p* = 0.001**	*p* = 0.068	***p* < 0.001**	1.0000

Above diagonal, magnitudes of correlation coefficient C; below diagonal, significance level *p* of correlation coefficient. Significant correlations are highlighted in bold. PL, proteasome location.

## Data Availability

The data presented in this study are available in Supplementary Material.

## Supplementary Materials

The following are available online: https://www.mdpi.com/2072-6694/13/5/1108/s1, Figure S1: specificity of antibodies to proteasome subunits and MHC class I molecules. Cleared homogenates of patient intestine parts (10 μL/90–105 μg of protein per lane) and rat thymus (7 μL/50 μg of protein per lane) were used. M, standard markers of molecular mass. Ab, antibodies, Figure S2: specificity of antibodies to subunits of proteasome activators and β actin. Cleared homogenates of patient intestine parts (10 μL/90–105 μg of protein per lane) were used. M, standard markers of molecular mass. Ab, antibodies, Table S1: distribution of proteasome activities in women with disease stage I, Table S2: distribution of proteasome activities in women with disease stage II, Table S3: distribution of proteasome activities in women with disease stage III, Table S4: distribution of proteasome activities in men with disease stage I, Table S5: distribution of proteasome activities in men with disease stage II, Table S6: distribution of proteasome activities in men with disease stage III, Table S7: tests of variance homogeneity. Effect of grouping factors “Patient gender⋅D. stage”, Table S8: tests of variance homogeneity. Effect of grouping factor “Patient gender”, Table S9: tests of variance homogeneity. Effect of grouping factor “D. stage”, Table S10: univariate tests of significance for proteasome activities.
