# Supplementary material for: Proteasomes in Patient Rectal Cancer and Different Intestine Locations: Where Does Proteasome Pool Change?
Source: Cancers (Basel). 2021 Mar 5;13(5):1108. doi: 10.3390/cancers13051108 (PMC7961961; doi:10.3390/cancers13051108)
Supplement: Supplementary file 1 [file cancers-13-01108-s001.zip › proofed supp/Table S1.pdf]

Table S1. Distribution of proteasome activities in women with disease stage I.

Activity	Designation	Gender, women; D. stage, I					Test of normality (p); Interval number 10		
		Valid N	Mean	Min	Max	St. D.	K-S test	Lill. test	S-W test
ChTL	(1)	6	23.88	18.40	32.00	5.04	>0.20	>0.20	0.727
	(2)	6	21.75	17.00	26.00	3.03	>0.20	>0.20	0.975
	(3)	6	6.55	3.90	8.60	2.12	>0.20	>0.20	0.111
	(4)	6	7.50	4.80	12.20	2.83	>0.20	>0.20	0.453
	(5)	6	6.75	4.00	11.70	2.86	>0.20	>0.20	0.299
	(6)	4	8.75	4.80	12.70	3.53	>0.20	>0.20	0.830
	(7)	3	6.13	3.60	9.20	2.84	>0.20	>0.20	0.688
CL	(1)	6	6.77	5.40	8.40	1.05	>0.20	>0.20	0.859
	(2)	6	2.65	2.30	3.10	0.31	>0.20	>0.20	0.755
	(3)	6	1.75	1.60	2.00	0.18	>0.20	<0.10	0.111
	(4)	6	1.83	1.30	2.40	0.43	>0.20	>0.20	0.724
	(5)	6	2.13	1.90	2.30	0.15	>0.20	<0.05	0.212
	(6)	4	2.03	1.70	2.30	0.32	>0.20	<0.20	0.100
	(7)	3	2.40	2.20	2.70	0.26	>0.20	<0.20	0.363
LMP7	(1)	3	13.50	8.50	18.00	4.77	>0.20	>0.20	0.826
	(2)	3	7.63	5.40	11.00	2.97	>0.20	<0.20	0.356
	(3)	3	3.10	1.20	5.40	2.13	>0.20	>0.20	0.688
	(4)	3	4.07	2.10	7.20	2.74	>0.20	<0.15	0.280
	(5)	3	5.90	3.90	8.20	2.17	>0.20	>0.20	0.770
	(6)	2	4.50	1.70	7.30	3.96	>0.20	>0.20	--
	(7)	2	4.10	1.70	6.50	3.39	>0.20	>0.20	--
LMP2	(1)	3	3.80	2.90	4.70	0.90	>0.20	>0.20	1.000
	(2)	3	2.43	1.70	2.90	0.64	>0.20	<0.15	0.298
	(3)	3	1.00	0.20	1.70	0.75	>0.20	>0.20	0.780
	(4)	3	0.63	0.40	0.80	0.21	>0.20	>0.20	0.463
	(5)	3	0.60	0.20	0.90	0.36	>0.20	>0.20	0.537
	(6)	2	1.40	0.90	1.90	0.71	>0.20	>0.20	--
	(7)	2	0.95	0.60	1.30	0.49	>0.20	>0.20	--

St. D., Standard deviation; K-S test, Kolmogorov-Smirnov test; Lill. test, Lilliefors test; S-W test, Shapiro-Wilk test.
