# Supplementary material for: Proteasomes in Patient Rectal Cancer and Different Intestine Locations: Where Does Proteasome Pool Change?
Source: Cancers (Basel). 2021 Mar 5;13(5):1108. doi: 10.3390/cancers13051108 (PMC7961961; doi:10.3390/cancers13051108)
Supplement: Supplementary file 1 [file cancers-13-01108-s001.zip › proofed supp/Table S10.pdf]

Table S10. Univariate tests of significance for proteasome activities.

Activity	Effect	Statistical indicators				
		SS	df	MS	F	p
ChTL	Intercept	40612.72	1	40612.72	3042.614	0.000000
	Patient gender	11.03	1	11.03	0.826	0.364057
	D.stage	75.06	2	37.53	2.812	0.061538
	Location	22174.24	6	3695.71	276.874	0.000000
	Location·D.stage	78.33	12	6.53	0.489	0.920820
	Location·Patient gender	67.73	6	11.29	0.846	0.535378
	D.stage·Patient gender	65.38	2	32.69	2.449	0.087944
	Error	4418.18	331	13.35		
CL	Intercept	2243.489	1	2243.489	7116.008	0.000000
	Patient gender	0.066	1	0.066	0.208	0.648764
	D.stage	0.251	2	0.126	0.399	0.671463
	Location	783.567	6	130.595	414.226	0.000000
	Patient gender·D.stage	0.325	2	0.162	0.515	0.597736
	Patient gender·Location	0.905	6	0.151	0.478	0.824269
	D.stage·Location	3.916	12	0.326	1.035	0.415900
	Error	104.356	331	0.315		
LMP7	Intercept	4876.532	1	4876.532	1069.339	0.000000
	Patient gender	19.567	1	19.567	4.291	0.039977
	D.stage	8.750	2	4.375	0.959	0.385395
	Location	2108.797	6	351.466	77.070	0.000000
	Patient gender·D.stage	41.030	2	20.515	4.499	0.012615
	Patient gender·Location	13.252	6	2.209	0.484	0.819319
	D.stage·Location	27.299	12	2.275	0.499	0.912978
	Error	706.850	155	4.560		
LMP2	Intercept	297.6682	1	297.6682	634.9000	0.000000
	Patient gender	0.2896	1	0.2896	0.6177	0.433106
	D.stage	0.8500	2	0.4250	0.9065	0.406076
	Location	165.6326	6	27.6054	58.8800	0.000000
	Patient gender·D.stage	0.1049	2	0.0524	0.1119	0.894235
	Patient gender·Location	1.7349	6	0.2892	0.6167	0.716683
	D.stage·Location	3.4618	12	0.2885	0.6153	0.826988
	Error	72.6706	155	0.4688		
