# Supplementary material for: Proteasomes in Patient Rectal Cancer and Different Intestine Locations: Where Does Proteasome Pool Change?
Source: Cancers (Basel). 2021 Mar 5;13(5):1108. doi: 10.3390/cancers13051108 (PMC7961961; doi:10.3390/cancers13051108)
Supplement: Supplementary file 1 [file cancers-13-01108-s001.zip › proofed supp/Table S2.pdf]

**Table S2.** Distribution of proteasome activities in women with disease stage II.

| Activity | Designation | Gender, women; D. stage, II | | | | | Test of normality (p); Interval number 10 | | |
|---|---|---|---|---|---|---|---|---|---|
| | | Valid N | Mean | Min | Max | St. D. | K-S test | Lill. test | S-W test |
| ChTL | (1) | 14 | 27.86 | 18.50 | 35.80 | 4.98 | >0.20 | >0.20 | 0.469 |
| | (2) | 14 | 19.76 | 14.10 | 27.90 | 4.00 | >0.20 | >0.20 | 0.129 |
| | (3) | 14 | 6.15 | 4.00 | 8.40 | 1.25 | >0.20 | >0.20 | 0.908 |
| | (4) | 14 | 7.19 | 4.00 | 11.40 | 1.86 | >0.20 | >0.20 | 0.717 |
| | (5) | 14 | 4.99 | 2.00 | 7.40 | 1.33 | >0.20 | >0.20 | 0.870 |
| | (6) | 12 | 8.26 | 4.20 | 14.90 | 3.02 | >0.20 | <0.05 | 0.112 |
| | (7) | 11 | 6.74 | 4.50 | 10.50 | 1.68 | >0.20 | <0.20 | 0.405 |
| CL | (1) | 14 | 6.16 | 5.20 | 7.90 | 0.83 | >0.20 | <0.15 | 0.213 |
| | (2) | 14 | 2.99 | 2.10 | 3.80 | 0.60 | >0.20 | >0.20 | 0.230 |
| | (3) | 14 | 1.85 | 1.50 | 2.30 | 0.27 | >0.20 | <0.10 | 0.173 |
| | (4) | 14 | 1.83 | 1.20 | 2.40 | 0.39 | >0.20 | <0.20 | 0.274 |
| | (5) | 14 | 2.24 | 1.60 | 2.70 | 0.35 | >0.20 | >0.20 | 0.460 |
| | (6) | 12 | 1.89 | 1.30 | 2.50 | 0.32 | >0.20 | >0.20 | 0.989 |
| | (7) | 11 | 2.17 | 1.60 | 3.20 | 0.53 | >0.20 | <0.20 | 0.096 |
| LMP7 | (1) | 7 | 13.11 | 10.10 | 16.90 | 2.73 | >0.20 | <0.20 | 0.166 |
| | (2) | 7 | 7.59 | 4.90 | 11.00 | 2.65 | >0.20 | <0.20 | 0.102 |
| | (3) | 7 | 3.74 | 0.50 | 5.80 | 1.95 | >0.20 | >0.20 | 0.588 |
| | (4) | 7 | 2.80 | 1.00 | 5.80 | 1.81 | >0.20 | >0.20 | 0.253 |
| | (5) | 7 | 4.27 | 1.30 | 7.10 | 2.02 | >0.20 | >0.20 | 0.781 |
| | (6) | 6 | 2.72 | 1.60 | 4.20 | 0.92 | >0.20 | >0.20 | 0.769 |
| | (7) | 5 | 3.26 | 1.60 | 5.40 | 1.40 | >0.20 | >0.20 | 0.812 |
| LMP2 | (1) | 7 | 3.26 | 1.70 | 4.80 | 1.18 | >0.20 | >0.20 | 0.585 |
| | (2) | 7 | 1.97 | 1.00 | 2.80 | 0.61 | >0.20 | >0.20 | 0.920 |
| | (3) | 7 | 0.33 | 0.10 | 0.90 | 0.29 | >0.20 | <0.20 | 0.055 |
| | (4) | 7 | 0.73 | 0.20 | 1.30 | 0.48 | >0.20 | <0.05 | 0.042 |
| | (5) | 7 | 0.89 | 0.20 | 1.40 | 0.42 | >0.20 | >0.20 | 0.800 |
| | (6) | 6 | 1.20 | 0.30 | 2.20 | 0.77 | >0.20 | >0.20 | 0.436 |
| | (7) | 5 | 0.70 | 0.30 | 1.50 | 0.47 | >0.20 | <0.15 | 0.168 |

St. D., Standard deviation; K-S test, Kolmogorov-Smirnov test; Lill. test, Lilliefors test; S-W test, Shapiro-Wilk test.
