# Supplementary material for: Proteasomes in Patient Rectal Cancer and Different Intestine Locations: Where Does Proteasome Pool Change?
Source: Cancers (Basel). 2021 Mar 5;13(5):1108. doi: 10.3390/cancers13051108 (PMC7961961; doi:10.3390/cancers13051108)
Supplement: Supplementary file 1 [file cancers-13-01108-s001.zip › proofed supp/Table S3.pdf]

Table S3. Distribution of proteasome activities in women with disease stage III.

Activity	Designation	Gender, women; D. stage, III					Test of normality (p); Interval number 10		
		Valid N	Mean	Min	Max	St. D.	K-S test	Lill. test	S-W test
ChTL	(1)	9	29.76	18.40	42.00	8.18	>0.20	>0.20	0.753
	(2)	9	18.97	11.00	23.00	3.94	>0.20	>0.20	0.233
	(3)	9	5.72	2.80	7.80	1.81	>0.20	>0.20	0.214
	(4)	9	7.59	4.00	12.50	2.82	>0.20	>0.20	0.681
	(5)	9	5.93	3.80	9.90	1.92	>0.20	<0.15	0.194
	(6)	9	7.87	5.10	10.60	1.74	>0.20	>0.20	0.973
	(7)	9	5.51	3.80	7.80	1.28	>0.20	>0.20	0.583
CL	(1)	9	6.67	5.00	8.00	1.02	>0.20	>0.20	0.378
	(2)	9	3.01	2.10	4.00	0.82	>0.20	<0.15	0.035
	(3)	9	1.81	1.30	2.30	0.36	>0.20	>0.20	0.620
	(4)	9	1.60	1.10	2.30	0.38	>0.20	>0.20	0.785
	(5)	9	2.06	1.40	2.70	0.46	>0.20	>0.20	0.616
	(6)	9	1.86	1.30	2.30	0.36	>0.20	>0.20	0.510
	(7)	9	2.01	1.20	2.50	0.42	>0.20	>0.20	0.309
LMP7	(1)	6	14.85	11.20	17.90	2.90	>0.20	>0.20	0.249
	(2)	6	8.22	5.40	10.30	2.21	>0.20	<0.15	0.123
	(3)	6	3.48	1.80	5.30	1.34	>0.20	>0.20	0.817
	(4)	6	3.28	0.80	6.50	2.60	>0.20	>0.20	0.144
	(5)	6	5.20	3.70	7.10	1.21	>0.20	>0.20	0.935
	(6)	6	2.90	0.80	4.50	1.28	>0.20	>0.20	0.665
	(7)	6	5.02	2.50	7.80	2.15	>0.20	>0.20	0.288
LMP2	(1)	6	3.75	1.90	5.20	1.18	>0.20	>0.20	0.949
	(2)	6	2.42	1.30	3.50	0.90	>0.20	>0.20	0.435
	(3)	6	0.55	0.10	1.10	0.42	>0.20	>0.20	0.334
	(4)	6	0.63	0.10	1.30	0.46	>0.20	>0.20	0.801
	(5)	6	1.07	0.10	1.60	0.57	>0.20	>0.20	0.315
	(6)	6	0.90	0.30	2.00	0.72	>0.20	<0.20	0.081
	(7)	6	0.43	0.10	1.00	0.35	>0.20	>0.20	0.292

St. D., Standard deviation; K-S test, Kolmogorov-Smirnov test; Lill. test, Lilliefors test; S-W test, Shapiro-Wilk test.
