# Supplementary material for: Proteasomes in Patient Rectal Cancer and Different Intestine Locations: Where Does Proteasome Pool Change?
Source: Cancers (Basel). 2021 Mar 5;13(5):1108. doi: 10.3390/cancers13051108 (PMC7961961; doi:10.3390/cancers13051108)
Supplement: Supplementary file 1 [file cancers-13-01108-s001.zip › proofed supp/Table S4.pdf]

Table S4. Distribution of proteasome activities in men with disease stage I.

Activity	Designation	Gender, men; D. stage, I					Test of normality (p); Interval number 5		
		Valid N	Mean	Min	Max	St. D.	K-S test	Lill. test	S-W test
ChTL	(1)	5	30.40	22.90	39.90	6.68	>0.20	>0.20	0.646
	(2)	5	20.08	6.90	32.60	9.77	>0.20	>0.20	0.986
	(3)	5	5.86	3.90	8.00	1.76	>0.20	>0.20	0.502
	(4)	5	7.06	4.20	8.80	2.04	>0.20	<0.15	0.178
	(5)	5	6.24	3.00	9.70	2.74	>0.20	>0.20	0.632
	(6)	3	6.43	3.20	10.80	3.92	>0.20	>0.20	0.517
	(7)	2	4.40	3.90	4.90	0.71	>0.20	>0.20	--
CL	(1)	5	6.18	5.50	6.80	0.51	>0.20	>0.20	0.980
	(2)	5	3.04	2.40	3.70	0.56	>0.20	>0.20	0.622
	(3)	5	1.88	1.50	2.20	0.31	>0.20	>0.20	0.332
	(4)	5	1.56	1.30	1.80	0.21	>0.20	>0.20	0.754
	(5)	5	2.16	1.70	2.60	0.34	>0.20	>0.20	0.994
	(6)	3	2.13	1.90	2.50	0.32	>0.20	<0.15	0.298
	(7)	2	2.65	2.40	2.90	0.35	>0.20	>0.20	--
LMP7	(1)	4	11.43	7.90	14.50	2.79	>0.20	>0.20	0.963
	(2)	4	8.28	6.70	11.00	1.88	>0.20	<0.15	0.206
	(3)	4	3.33	1.00	4.60	1.68	>0.20	>0.20	0.256
	(4)	4	2.63	0.60	7.10	3.06	>0.20	<0.20	0.079
	(5)	4	3.55	1.20	5.60	2.09	>0.20	>0.20	0.475
	(6)	2	4.95	1.00	8.90	5.59	>0.20	>0.20	--
	(7)	1	5.40	5.40	5.40		--	--	--
LMP2	(1)	4	3.30	1.40	5.80	1.86	>0.20	>0.20	0.792
	(2)	4	1.90	1.10	3.10	0.91	>0.20	>0.20	0.517
	(3)	4	0.58	0.30	0.90	0.28	>0.20	>0.20	0.650
	(4)	4	0.88	0.50	1.20	0.30	>0.20	>0.20	0.952
	(5)	4	0.90	0.30	1.50	0.59	>0.20	>0.20	0.348
	(6)	2	1.35	1.30	1.40	0.07	>0.20	>0.20	--
	(7)	1	0.50	0.50	0.50		--	--	--

St. D., Standard deviation; K-S test, Kolmogorov-Smirnov test; Lill. test, Lilliefors test; S-W test, Shapiro-Wilk test.
