# Supplementary material for: Proteasomes in Patient Rectal Cancer and Different Intestine Locations: Where Does Proteasome Pool Change?
Source: Cancers (Basel). 2021 Mar 5;13(5):1108. doi: 10.3390/cancers13051108 (PMC7961961; doi:10.3390/cancers13051108)
Supplement: Supplementary file 1 [file cancers-13-01108-s001.zip › proofed supp/Table S5.pdf]

Table S5. Distribution of proteasome activities in men with disease stage II.

Activity	Designation	Gender, men; D. stage, II					Test of normality (p); Interval number 10		
		Valid N	Mean	Min	Max	St. D.	K-S test	Lill. test	S-W test
ChTL	(1)	12	28.67	18.00	37.00	6.15	>0.20	>0.20	0.714
	(2)	12	19.24	12.80	35.70	6.24	>0.20	<0.01	0.010
	(3)	12	5.93	4.20	8.00	1.39	>0.20	>0.20	0.193
	(4)	12	6.78	4.00	10.00	2.18	>0.20	>0.20	0.224
	(5)	12	5.55	2.70	7.90	1.79	>0.20	>0.20	0.403
	(6)	12	6.30	3.00	9.20	1.99	>0.20	>0.20	0.725
	(7)	10	5.49	2.40	8.90	1.83	>0.20	>0.20	0.729
CL	(1)	12	6.25	4.90	8.60	1.13	>0.20	>0.20	0.203
	(2)	12	3.02	2.30	4.00	0.59	>0.20	>0.20	0.229
	(3)	12	1.93	1.40	2.40	0.39	>0.20	>0.20	0.044
	(4)	12	1.87	1.40	3.00	0.46	>0.20	<0.10	0.062
	(5)	12	1.93	1.50	2.60	0.41	>0.20	<0.05	0.020
	(6)	12	1.98	1.00	2.50	0.49	>0.20	>0.20	0.177
	(7)	10	2.10	0.90	2.80	0.62	>0.20	>0.20	0.361
LMP7	(1)	4	15.08	11.60	17.00	2.39	>0.20	<0.15	0.200
	(2)	4	8.50	5.70	12.20	2.71	>0.20	>0.20	0.584
	(3)	4	3.55	2.10	5.60	1.52	>0.20	>0.20	0.701
	(4)	4	2.33	0.90	5.60	2.20	>0.20	<0.05	0.027
	(5)	4	4.23	2.10	5.20	1.43	>0.20	<0.10	0.049
	(6)	4	3.38	1.70	4.70	1.24	>0.20	>0.20	0.615
	(7)	3	3.10	1.80	5.40	2.00	>0.20	<0.10	0.143
LMP2	(1)	4	2.98	1.70	4.50	1.19	>0.20	>0.20	0.931
	(2)	4	1.70	1.10	2.00	0.41	>0.20	<0.15	0.115
	(3)	4	0.50	0.20	1.00	0.35	>0.20	<0.10	0.194
	(4)	4	0.88	0.70	1.20	0.24	>0.20	>0.20	0.220
	(5)	4	0.90	0.30	1.60	0.61	>0.20	>0.20	0.578
	(6)	4	0.90	0.40	1.30	0.39	>0.20	>0.20	0.850
	(7)	3	0.93	0.50	1.20	0.38	>0.20	<0.15	0.253

St. D., Standard deviation; K-S test, Kolmogorov-Smirnov test; Lill. test, Lilliefors test; S-W test, Shapiro-Wilk test.
