# Supplementary material for: Proteasomes in Patient Rectal Cancer and Different Intestine Locations: Where Does Proteasome Pool Change?
Source: Cancers (Basel). 2021 Mar 5;13(5):1108. doi: 10.3390/cancers13051108 (PMC7961961; doi:10.3390/cancers13051108)
Supplement: Supplementary file 1 [file cancers-13-01108-s001.zip › proofed supp/Table S6.pdf]

Table S6. Distribution of proteasome activities in men with disease stage III.

Activity	Designation	Gender, men; D. stage, III					Test of normality (p); Interval number 10		
		Valid N	Mean	Min	Max	St. D.	K-S test	Lill. test	S-W test
ChTL	(1)	8	30.59	24.70	40.20	5.76	>0.20	>0.20	0.121
	(2)	8	22.20	12.30	28.40	5.80	>0.20	>0.20	0.453
	(3)	8	7.18	4.40	8.80	1.68	>0.20	<0.05	0.099
	(4)	8	9.30	5.20	14.40	3.09	>0.20	>0.20	0.907
	(5)	8	6.31	3.40	9.00	1.91	>0.20	>0.20	0.887
	(6)	8	8.13	5.30	14.50	3.37	>0.20	<0.05	0.051
	(7)	8	8.14	3.70	12.30	2.67	>0.20	>0.20	0.999
CL	(1)	8	6.68	5.80	8.10	0.94	>0.20	>0.20	0.045
	(2)	8	2.90	2.20	3.70	0.61	>0.20	>0.20	0.208
	(3)	8	2.24	1.60	3.00	0.48	>0.20	>0.20	0.779
	(4)	8	1.85	1.40	3.20	0.60	>0.20	>0.20	0.013
	(5)	8	2.11	1.60	2.60	0.38	>0.20	>0.20	0.363
	(6)	8	1.93	1.40	3.30	0.67	>0.20	>0.20	0.047
	(7)	8	2.13	1.30	2.90	0.54	>0.20	>0.20	0.705
LMP7	(1)	4	10.55	9.80	12.30	1.18	>0.20	>0.20	0.038
	(2)	4	6.48	5.20	7.50	1.15	>0.20	>0.20	0.244
	(3)	4	2.18	0.50	3.90	1.59	>0.20	>0.20	0.579
	(4)	4	2.45	1.10	4.50	1.55	>0.20	>0.20	0.486
	(5)	4	3.35	1.80	4.90	1.27	>0.20	>0.20	0.903
	(6)	4	2.63	1.10	3.70	1.15	>0.20	>0.20	0.658
	(7)	4	2.68	1.40	4.20	1.17	>0.20	>0.20	0.909
LMP2	(1)	4	3.40	1.90	4.50	1.23	>0.20	>0.20	0.444
	(2)	4	2.20	1.20	2.90	0.84	>0.20	>0.20	0.237
	(3)	4	0.55	0.20	1.10	0.39	>0.20	<0.20	0.414
	(4)	4	0.60	0.10	0.90	0.36	>0.20	>0.20	0.405
	(5)	4	0.85	0.40	1.70	0.61	>0.20	>0.20	0.207
	(6)	4	0.88	0.20	1.30	0.50	>0.20	>0.20	0.457
	(7)	4	0.90	0.30	1.40	0.45	>0.20	>0.20	0.797

St. D., Standard deviation; K-S test, Kolmogorov-Smirnov test; Lill. test, Lilliefors test; S-W test, Shapiro-Wilk test.
