# Supplementary material for: Proteasomes in Patient Rectal Cancer and Different Intestine Locations: Where Does Proteasome Pool Change?
Source: Cancers (Basel). 2021 Mar 5;13(5):1108. doi: 10.3390/cancers13051108 (PMC7961961; doi:10.3390/cancers13051108)
Supplement: Supplementary file 1 [file cancers-13-01108-s001.zip › proofed supp/Table S7.pdf]

Table S7. Tests of variance homogeneity. Effect of grouping factors "Patient gender·D. stage".

Activity	Designation	Hartley F-max	Cochran C	Bartlett Chi--Sqr	df	p
ChTL	(1)	4.44	0.36	2.17	4	0.705
	(2)	141.3	0.54	5.23	4	0.265
	(3)		0.31	–0.24	3	1.000
	(4)	5.54	0.49	2.96	4	0.565
	(5)	7.04	0.38	3.53	4	0.473
	(6)	28.56	0.53	6.50	4	0.165
	(7)	8.34	0.36	4.11	4	0.391
CL	(1)	8.63	0.33	3.03	4	0.553
	(2)	13.84	0.31	1.64	4	0.802
	(3)	32.50	0.37	3.04	4	0.551
	(4)	18.06	0.56	4.26	4	0.372
	(5)		0.34	–0.47	3	1.000
	(6)	84.92	0.69	9.32	4	0.054
	(7)	13.63	0.55	3.74	4	0.442
LMP7	(1)	32.46	0.67	5.08	4	0.279
	(2)	11.80	0.51	3.18	4	0.527
	(3)	13.71	0.59	2.84	4	0.585
	(4)	139.3	0.54	7.43	4	0.115
	(5)	6.33	0.50	2.07	4	0.724
	(6)	22.63	0.77	5.83	4	0.212
	(7)	8.41	0.49	2.43	4	0.658
LMP2	(1)	5.14	0.31	0.61	4	0.962
	(2)	40.68	0.35	2.86	4	0.582
	(3)	1.68	0.23	0.27	4	0.991
	(4)	4.77	0.34	1.32	4	0.858
	(5)	18.83	0.34	3.43	4	0.489
	(6)	2.33	0.26	0.74	4	0.947
	(7)	2.00	0.26	0.47	4	0.976
