# Supplementary material for: Proteasomes in Patient Rectal Cancer and Different Intestine Locations: Where Does Proteasome Pool Change?
Source: Cancers (Basel). 2021 Mar 5;13(5):1108. doi: 10.3390/cancers13051108 (PMC7961961; doi:10.3390/cancers13051108)
Supplement: Supplementary file 1 [file cancers-13-01108-s001.zip › proofed supp/Table S8.pdf]

Table S8. Tests of variance homogeneity. Effect of grouping factor "Patient gender".

Activity	Designation	Hartley F-max	Cochran C	Bartlett Chi--Sqr	df	p
ChTL	(1)	1.15	0.54	0.04	1	0.839
	(2)	3.12	0.76	2.85	1	0.092
	(3)	1.35	0.57	0.18	1	0.673
	(4)	2.15	0.68	1.28	1	0.257
	(5)	3.25	0.76	3.05	1	0.081
	(6)	1.44	0.59	0.29	1	0.591
	(7)	1.24	0.55	0.10	1	0.755
CL	(1)	1.39	0.58	0.21	1	0.643
	(2)	1.52	0.60	0.35	1	0.554
	(3)	1.48	0.60	0.33	1	0.568
	(4)	2.02	0.67	1.07	1	0.300
	(5)	1.74	0.63	0.60	1	0.438
	(6)	4.03	0.80	4.24	1	0.040
	(7)	2.77	0.73	2.27	1	0.132
LMP7	(1)	1.03	0.51	0.00	1	0.962
	(2)	4.57	0.82	3.95	1	0.047
	(3)	1.40	0.58	0.23	1	0.634
	(4)	3.04	0.75	2.26	1	0.133
	(5)	1.49	0.60	0.31	1	0.575
	(6)	1.97	0.66	0.90	1	0.344
	(7)	1.65	0.62	0.49	1	0.482
LMP2	(1)	1.72	0.63	0.64	1	0.425
	(2)	1.05	0.51	0.00	1	0.948
	(3)	2.16	0.68	1.14	1	0.285
	(4)	1.26	0.56	0.11	1	0.743
	(5)	1.63	0.62	0.51	1	0.475
	(6)	2.55	0.72	1.64	1	0.201
	(7)	1.22	0.55	0.08	1	0.775
