# Supplementary material for: Proteasomes in Patient Rectal Cancer and Different Intestine Locations: Where Does Proteasome Pool Change?
Source: Cancers (Basel). 2021 Mar 5;13(5):1108. doi: 10.3390/cancers13051108 (PMC7961961; doi:10.3390/cancers13051108)
Supplement: Supplementary file 1 [file cancers-13-01108-s001.zip › proofed supp/Table S9.pdf]

Table S9. Tests of variance homogeneity. Effect of grouping factor "D. stage".

Activity	Designation	Hartley F-max	Cochran C	Bartlett Chi--Sqr	df	p
ChTL	(1)	3.56	0.63	2.99	2	0.225
	(2)	30.27	0.58	4.18	2	0.124
	(3)	3.70	0.55	1.32	2	0.516
	(4)	3.45	0.56	1.49	2	0.474
	(5)	4.46	0.64	2.15	2	0.341
	(6)	7.64	0.50	1.92	2	0.382
	(7)	1.39	0.40	0.08	2	0.960
CL	(1)	1.54	0.40	0.21	2	0.898
	(2)	22.33	0.52	3.69	2	0.158
	(3)	4.46	0.62	2.33	2	0.312
	(4)	3.51	0.49	2.50	2	0.286
	(5)	14.87	0.54	3.01	2	0.222
	(6)	3.83	0.64	2.83	2	0.243
	(7)	4.74	0.57	1.59	2	0.452
LMP7	(1)	6.19	0.68	2.94	2	0.230
	(2)	2.17	0.49	0.51	2	0.774
	(3)	3.44	0.60	1.35	2	0.510
	(4)	7.94	0.66	3.90	2	0.142
	(5)	2.21	0.49	0.55	2	0.761
	(6)	16.50	0.85	8.41	2	0.015
	(7)	2.79	0.48	1.11	2	0.575
LMP2	(1)	1.37	0.37	0.06	2	0.968
	(2)	17.56	0.62	3.59	2	0.166
	(3)	2.17	0.49	0.47	2	0.789
	(4)	1.03	0.34	0.00	2	0.999
	(5)	3.40	0.58	1.80	2	0.407
	(6)	1.58	0.39	0.14	2	0.935
	(7)	1.06	0.34	0.01	2	0.997
