# Supplementary figures and images for: Proteasomes in Patient Rectal Cancer and Different Intestine Locations: Where Does Proteasome Pool Change?
Source: Cancers (Basel). 2021 Mar 5;13(5):1108. doi: 10.3390/cancers13051108 (PMC7961961; doi:10.3390/cancers13051108)

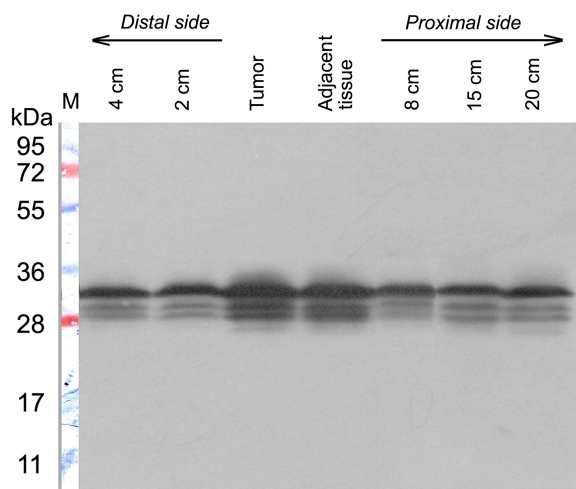

Mouse Ab to  $\alpha 1,2,3,5,6,7$

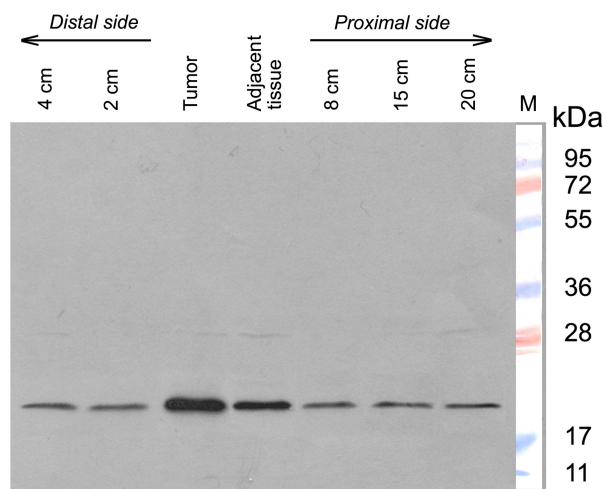

Mouse Ab to LMP2

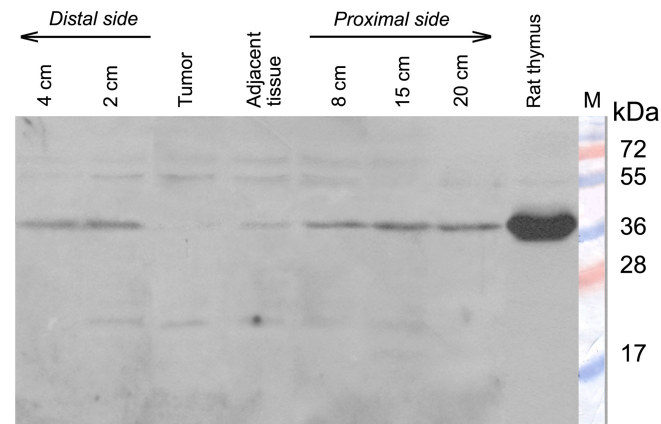

Rabbit Ab to MHC I

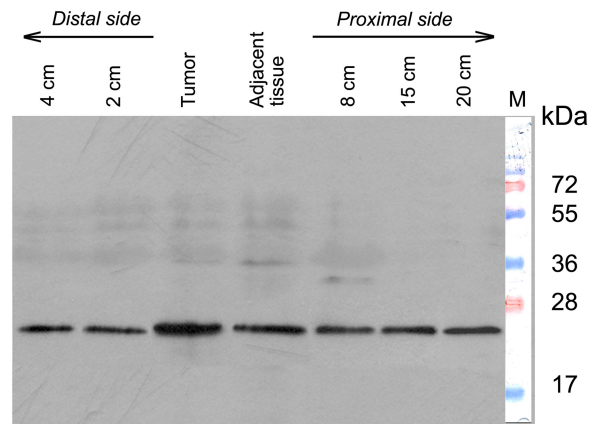

Mouse Ab to LMP7

Supplement: Supplementary file 1 [file cancers-13-01108-s001.zip › proofed supp/Fig. S1.pdf]

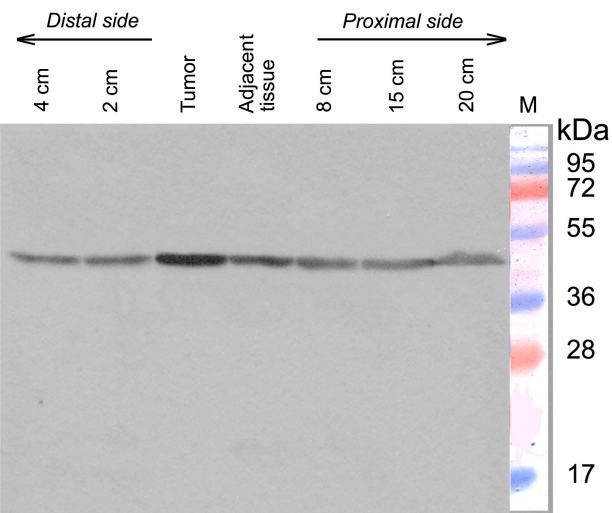

Mouse Ab to Rpt6

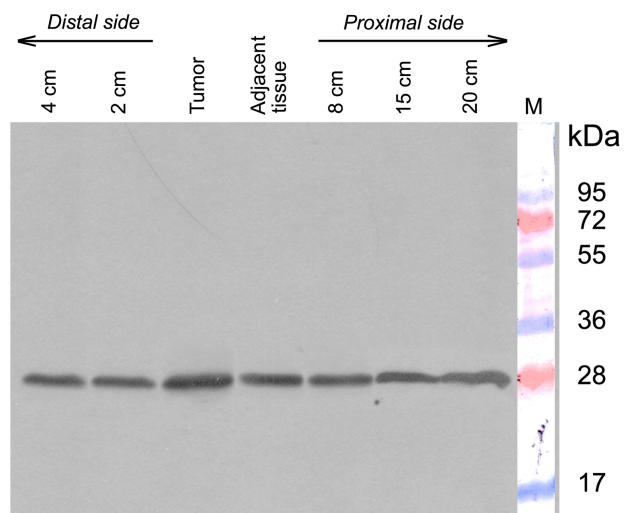

Rabbit Ab to PA28 alpha

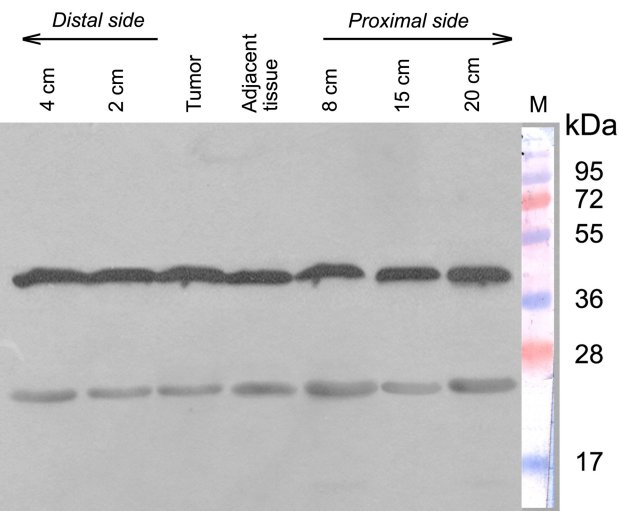

Mouse Ab to beta-actin

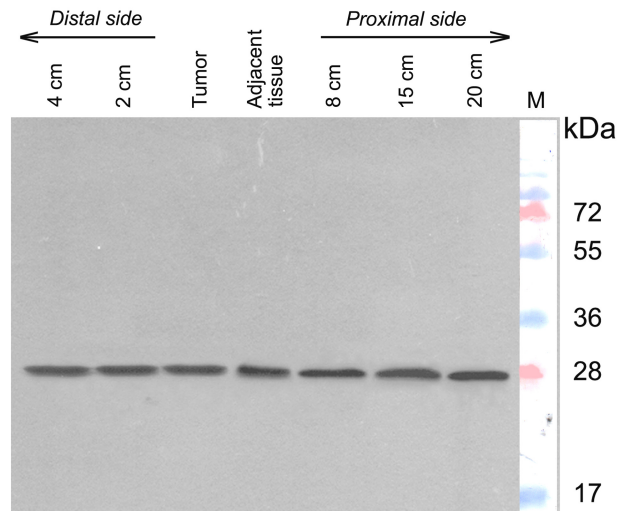

Rabbit Ab to PA28 gamma

Supplement: Supplementary file 1 [file cancers-13-01108-s001.zip › proofed supp/Fig. S2.pdf]

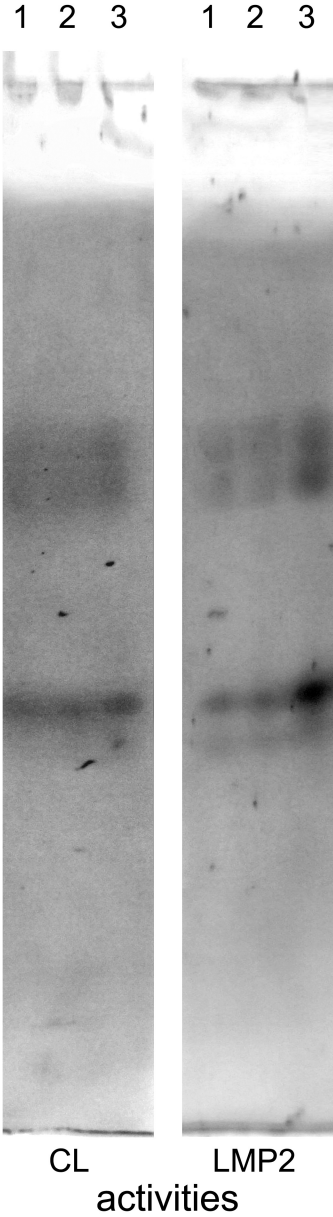

For Figure 4

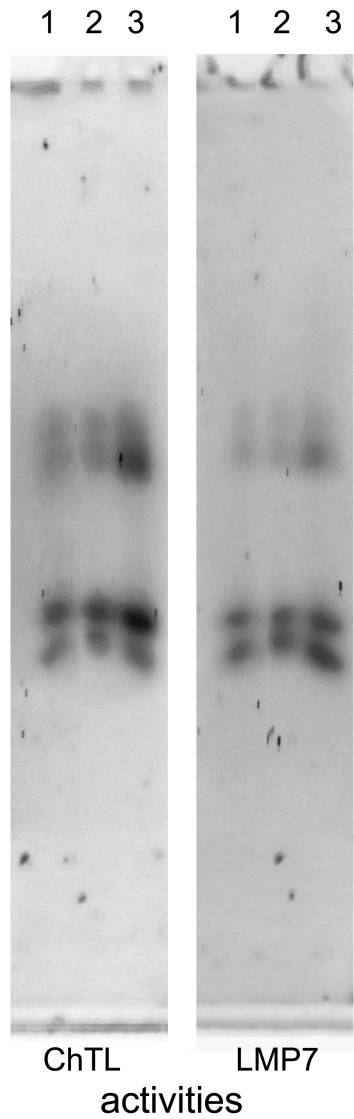

For Figure 5

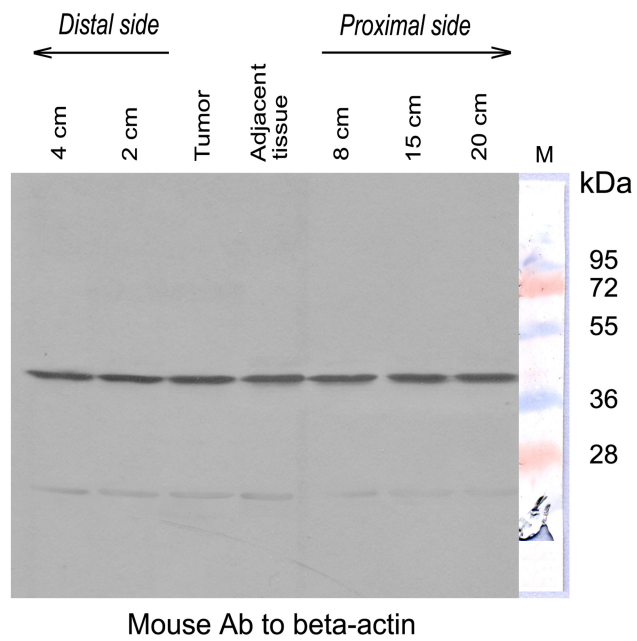

For Figure 2

Supplement: Supplementary file 1 [file cancers-13-01108-s001.zip › proofed supp/Gel images.pdf]
